# Influential articles in autism and gut microbiota: bibliometric profile and research trends

**DOI:** 10.3389/fmicb.2024.1401597

**Published:** 2025-01-09

**Authors:** Jiangbo Ying, Melvyn Weibin Zhang, Ker-Chiah Wei, Sunny H. Wong, Mythily Subramaniam

**Affiliations:** ^1^Department of Developmental Psychiatry, Institute of Mental Health, Singapore, Singapore; ^2^Central Region, Institute of Mental Health, Singapore, Singapore; ^3^Lee Kong Chian School of Medicine, Nanyang Technological University, Singapore, Singapore; ^4^Department of Gastroenterology and Hepatology, Tan Tock Seng Hospital, Singapore, Singapore; ^5^Research Division, Institute of Mental Health, Singapore, Singapore

**Keywords:** bibliometric analysis, autism, gut microbiota, research trends, citations

## Abstract

**Objective:**

Autism spectrum disorder (ASD) is a common neurodevelopmental disorder. Increasing evidence suggests that it is potentially related to gut microbiota, but no prior bibliometric analysis has been performed to explore the most influential works in the relationships between ASD and gut microbiota. In this study, we conducted an in-depth analysis of the most-cited articles in this field, aiming to provide insights to the existing body of research and guide future directions.

**Methods:**

A search strategy was constructed and conducted in the Web of Science database to identify the 100 most-cited papers in ASD and gut microbiota. The Biblioshiny package in R was used to analyze and visualize the relevant information, including citation counts, country distributions, authors, journals, and thematic analysis. Correlation and comparison analyses were performed using SPSS software.

**Results:**

The top 100 influential manuscripts were published between 2000 and 2021, with a total citation of 40,662. The average number of citations annually increased over the years and was significantly correlated to the year of publication (*r* = 0.481, *p* < 0.01, Spearman’s rho test). The United States was involved in the highest number of publications (*n* = 42). The number of publications in the journal was not significantly related to the journal’s latest impact factor (*r* = 0.016, *p* > 0.05, Spearman’s rho test). Co-occurrence network and thematic analysis identified several important areas, such as microbial metabolites of short-chain fatty acids and overlaps with irritable bowel syndrome.

**Conclusion:**

This bibliometric analysis provides the key information of the most influential studies in the area of ASD and gut microbiota, and suggests the hot topics and future directions. The findings of this study can serve as a valuable reference for researchers and policymakers, guiding the development and implementation of the scientific research strategies in this area.

## Introduction

Autism spectrum disorder (ASD) is a neurodevelopmental disorder characterized by deficits in social communication and interaction, alongside the manifestation of repetitive and restrictive behavior patterns (APA, 2022). The global prevalence of ASD has been estimated to be around 1%, and the prevalence estimates have increased over time in various countries (Zeidan et al., 2022). Persons with ASD may have emotional and behavioral problems, such as self-harm, aggression, temper tantrums, and property destruction (Jang et al., 2011). They often have other psychiatric conditions, such as anxiety, depression, and psychosis (Dan et al., 2020). The economic costs of ASD are huge, and they include costs for healthcare services, special education, production loss for persons with ASD, lost productivity for caregivers, and respite care (Rogge and Janssen, 2019). In the United States, it has been reported that the average yearly expenditure for emergency room services is $15,929 for ASD, compared to $2,598 for non-ASD; and yearly expenditure for outpatient visits is $4,375 for ASD compared to $824 for non-ASD (Vohra et al., 2017). In the United Kingdom, it has been estimated that adolescents with ASD who need additional special education or residential schooling can cost £10,507 in 6 months (Barrett et al., 2015).

The composition of the gut microbiota has been reported to be associated with ASD. The gut microbiota has a very diverse composition and is composed of bacteria, as well as fungi, viruses and protists (Enaud et al., 2018). It has a bidirectional connection with the central nervous system. Millions of nerve cells in the gut form the enteric nervous system which is recognized as a second brain (Gershon, 1999). The microbiota-gut-brain axis has been studied and the bidirectional communication of this pathway occurs through various mechanisms, including enteric nervous system, autonomic nervous system, immune system, hormones, and neurotransmitters (Cryan and Dinan, 2012). Possible involvement of a microbial element in the pathogenesis of ASD was first reported in 1998, when Bolte (1998) introduced the hypothesis that *Clostridium tetani* neurotoxin was transported from the gastrointestinal tract to the central nervous system via the vagus nerve, causing symptoms of ASD. The link between gut microbiota and ASD has been studied in animal models. One study published in 2019 that transplanted gut microbiota from human ASD patients into germ-free mice revealed development of hallmark autistic behaviors in the recipient animals (Sharon et al., 2019). The association between gut microbiota and ASD has also been reported in human studies. For example a pyrosequencing study observed that *Bacteroidetes* were present at high levels in the persons with ASD, while *Firmicutes* were more abundant in the healthy control group (Finegold et al., 2010).

Given the rising trend of interest related to ASD and gut microbiota, it is worthwhile to identify the most influential scientific achievements amidst the abundance of literature in this research area. Bibliometric analysis is a widely used, rigorous approach for exploring extensive scientific datasets and extracting useful information, such as author names, total citations, and country distributions (Donthu et al., 2021). It can visualize the detailed results and help researchers to develop a thorough understanding of the research trajectory in the field and identify research hotspots and gaps. For example, a recent bibliometric analysis presents a comprehensive global overview of artificial intelligence in life science research and suggests that coordinated international research efforts are necessary to advance this research area (Schmallenbach et al., 2024). As bibliometric analysis offers both quantitative and qualitative insights into the influence and evolution of academic communication, it assists policymakers to track emerging trends and make informed choices about research funding and collaboration strategies (Hassan and Duarte, 2024).

To the best of our knowledge, no prior bibliometric analysis has been performed to explore the most influential works in the field of ASD and gut microbiota. This study seeks to fill this gap by conducting an in-depth analysis of the most-cited articles concerning the intersection of ASD and gut microbiota, with the goal of providing valuable insights to the existing body of research and guiding researchers and policymakers in evaluating and making informed decisions related to this field.

## Materials and methods

### Article selection

The Clarivate Analytics Web of Science database was used to identify relevant articles in this current bibliometric review. The Web of Science database has the capability to retrieve numerous articles with comprehensive details, including titles, author names, total download times, and total citations. It is an extensive repository which includes major journals across more than 170 subjects (Quan et al., 2024). In addition, it enhances coverage by including citations from scientific publications dating back to 1900 and encompasses all significant high-impact scientific journals (Martin-Martin et al., 2018; Tomova et al., 2015). Various studies, including those on gut microbiota and other diseases, have relied solely on the Web of Science database as their primary source for conducting bibliometric analyses (Chang et al., 2023; Ring et al., 2020; Wan et al., 2022; Ying et al., 2022).

To find pertinent articles, several recent systematic reviews related to ASD or gut microbiota were referenced to create search terms (Lewandowska-Pietruszka et al., 2023; Perna et al., 2023; Wang A. et al., 2023). Besides, an information specialist was consulted to help in further refinement of the search strategies and ensure the comprehensive retrieval of all relevant articles. The following search terminologies were used in this bibliometric review: TS = (“autism” OR “autistic” OR “Asperger*” OR “pervasive developmental disorder*”) AND TS = (“microbiome*” OR “microbiota*” OR “flora*” OR “microbe*” OR “microflora*” OR “microbial”). The terminology TS denotes a search focused on the topic of interest.

Using this approach, the Web of Science database was systematically searched in January 2024. No restrictions were implemented in terms of the language of articles and the publication dates. The publications were ranked according to the number of citations, and they were then reviewed to identify the 100 most-cited papers. Studies were included if (1) one of their focuses was related to the topic of ASD and gut microbiota, (2) the type of the document was either Article or Review Article according to the Web of Science database. Other types of documents, such as Editorial Material, Meeting Abstract, and Book Chapters, were excluded. Two authors (JY and MZ) independently performed the selection of the top 100 papers with the most citations, based on the title and abstract and reading the full texts if needed. If any disagreement arose, a third author was consulted to achieve an agreement.

### Data analysis

The bibliometric data analysis was performed using Biblioshiny package in R (Version 4.3.2) (Aria and Cuccurullo, 2017). The Biblioshiny package was previously utilized for this type of analysis in various areas, such as the application of deep learning in cancer (Wang R. et al., 2024), the use of monoclonal antibodies for atherosclerosis (Ma et al., 2023), and the global impact of metaproteomics research (Ascandari et al., 2023). All data were downloaded from the Web of Science database and imported into Biblioshiny, which could convert and analyze the information, including the authors, years of publication, number of citations, and distribution of countries/regions. The study type was categorized into three main groups: (1) animal studies, which incorporated animal models in their study design; (2) human studies, which involved persons with ASD; and (3) reviews, encompassing literature reviews or systematic reviews. The impact factor of each journal was extracted from the Clarivate Analytics Journal Citation Reports.

All statistical analysis was conducted using SPSS software (Version 25). The Shapiro–Wilk test was used to test the normality of the distribution of variables. Spearman’s rho test was applied to assess the correlations between two variables. Mann–Whitney *U* test was performed to assess for any statistically significant differences between two groups, and the Kruskal–Wallis test was conducted to compare the differences between three or more groups. All *p* values were two-tailed, and a *p*-value of ≤0.05 was considered to indicate statistical significance.

## Results

### Overview

**FIGURE 1:**
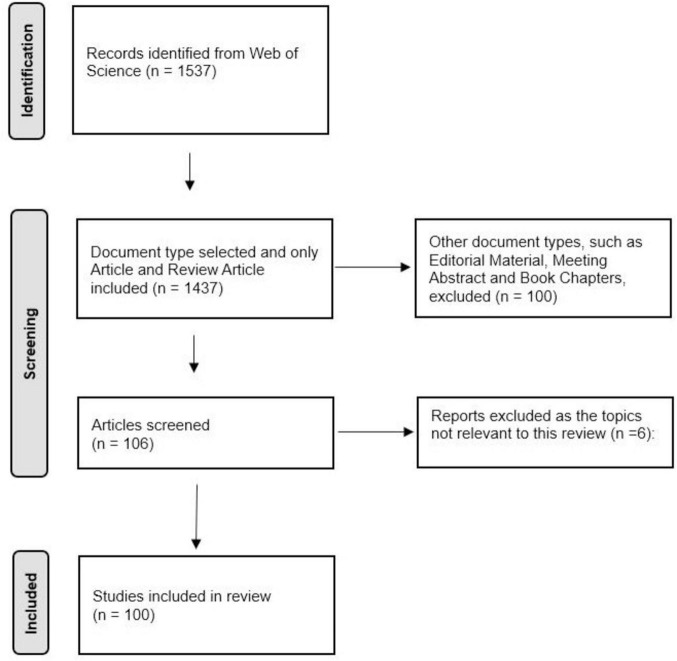
PRISMA flow diagram of the study selection process.

**FIGURE 2:**
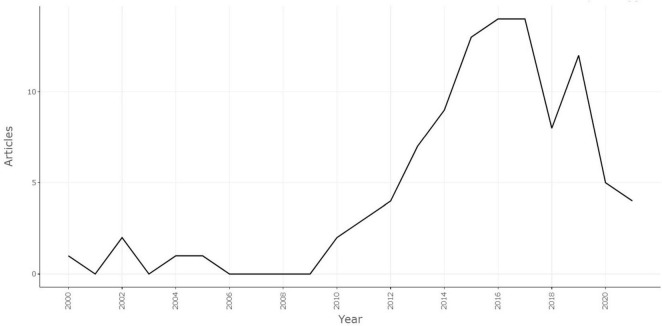
The trends of the annual publications of the 100 most-cited articles.

A total of 1,537 articles were retrieved from the Web of Science database on 10 January 2024, and the 100 most-cited papers were identified after screening. The Preferred Reporting Items for Systematic Reviews and Meta-Analyses (PRISMA) was used to describe the detailed screening process (Figure 1). General information of the selected articles is detailed in Table 1. The trends of the annual publications of the 100 most-cited articles are described in Figure 2.

**TABLE 1:** The 100 most-cited articles in autism spectrum disorder and gut microbiota.

No.	Reference	Journal	Journal impact factor at the year of publication	Journal impact factor in 2023	Article	Study type	Total citations	Annual citations
1	Hsiao et al., 2013	*Cell*	33.116	45.6	Microbiota modulate behavioral and physiological abnormalities associated with neurodevelopmental disorders	Animal study	2,081	173.42
2	Cryan et al., 2019	*Physiological Reviews*	25.588	33.4	The microbiota-gut-brain axis	Review	1,769	294.83
3	Carabotti et al., 2015	*Annals of Gastroenterology*	Nil	2.1	The gut-brain axis: interactions between enteric microbiota, central and enteric nervous systems	Review	1,412	141.20
4	Rinninella et al., 2019	*Microorganisms*	4.152	4.1	What is the healthy gut microbiota composition? A changing ecosystem across age, environment, diet, and diseases	Review	1,395	232.50
5	Gilbert et al., 2018	*Nature Medicine*	30.641	58.7	Current understanding of the human microbiome	Review	1,133	161.86
6	Fung et al., 2017	*Nature Neuroscience*	19.912	21.3	Interactions between the microbiota, immune and nervous systems in health and disease	Review	1,027	128.38
7	Riviere et al., 2016	*Frontiers in Microbiology*	4.076	4.0	Bifidobacteria and butyrate-producing colon bacteria: importance and strategies for their stimulation in the human gut	Review	928	103.11
8	Mayer et al., 2015	*Journal of Clinical Investigation*	12.575	13.3	Gut/brain axis and the microbiota	Review	847	84.70
9	Nguyen et al., 2015	*Disease Models & Mechanisms*	4.316	4.0	How informative is the mouse for human gut microbiota research?	Review	808	80.80
10	Sharon et al., 2016	*Cell*	30.41	45.6	The central nervous system and the gut microbiome	Review	787	87.44
11	Kang et al., 2017	*Microbiome*	9.133	13.8	Microbiota transfer therapy alters gut ecosystem and improves gastrointestinal and autism symptoms: an open-label study	Human study	722	90.25
12	Borre et al., 2014b	*Trends in Molecular Medicine*	9.453	12.8	Microbiota and neurodevelopmental windows: implications for brain disorders	Review	690	62.73
13	Buffington et al., 2016	*Cell*	30.41	45.6	Microbial reconstitution reverses maternal diet-induced social and synaptic deficits in offspring	Animal study	678	75.33
14	Sampson and Mazmanian, 2015	*Cell Host & Microbe*	12.552	20.6	Control of brain development, function, and behavior by the microbiome	Review	664	66.40
15	Morais et al., 2021	*Nature Reviews Microbiology*	78.297	69.2	The gut microbiota-brain axis in behaviour and brain disorders	Review	659	164.75
16	Finegold et al., 2010	*Anaerobe*	2.448	2.5	Pyrosequencing study of fecal microflora of autistic and control children	Human study	643	42.87
17	Adams et al., 2011	*BMC Gastroenterology*	2.422	2.5	Gastrointestinal flora and gastrointestinal status in children with autism-comparisons to typical children and correlation with autism severity	Human study	625	44.64
18	Kang et al., 2013	*PLoS One*	3.534	2.9	Reduced incidence of prevotella and other fermenters in intestinal microflora of autistic children	Human study	605	50.42
19	Mayer et al., 2014a	*Journal of Neuroscience*	6.344	4.4	Gut microbes and the brain: paradigm shift in neuroscience	Review	559	50.82
20	Sharon et al., 2019	*Cell*	38.637	45.6	Human gut microbiota from autism spectrum disorder promote behavioral symptoms in mice	Animal study	542	90.33
21	Strati et al., 2017	*Microbiome*	9.133	13.8	New evidences on the altered gut microbiota in autism spectrum disorders	Human study	539	67.38
22	Parracho et al., 2005	*Journal of Medical Microbiology*	2.318	2.4	Differences between the gut microflora of children with autistic spectrum disorders and that of healthy children	Human study	527	26.35
23	Dinan and Cryan, 2017c	*Gastroenterology Clinics of North America*	3.265	2.9	The microbiome-gut-brain axis in health and disease	Review	526	65.75
24	De Angelis et al., 2013	*PLoS One*	3.534	2.9	Fecal microbiota and metabolome of children with autism and pervasive developmental disorder not otherwise specified	Human study	526	43.83
25	Zhang et al., 2015	*International Journal of Molecular Sciences*	3.257	4.9	Impacts of gut bacteria on human health and diseases	Review	513	51.30
26	Kho and Lal, 2018	*Frontiers in Microbiology*	4.259	4.0	The human gut microbiome – a potential controller of wellness and disease	Review	511	73.00
27	Cryan et al., 2020	*Lancet Neurology*	44.182	46.6	The gut microbiome in neurological disorders	Review	503	100.60
28	Wang and Kasper, 2014	*Brain Behavior and Immunity*	5.889	8.8	The role of microbiome in central nervous system disorders	Review	500	45.45
29	Hills et al., 2019	*Nutrients*	4.546	4.8	Gut microbiome: profound implications for diet and disease	Review	488	81.33
30	Finegold et al., 2002	*Clinical Infectious Diseases*	Nil	8.2	Gastrointestinal microflora studies in late-onset autism	Human study	476	20.70
31	Dinan and Cryan, 2017b	*Journal of Physiology-London*	4.54	4.7	Gut instincts: microbiota as a key regulator of brain development, ageing and neurodegeneration	Review	431	53.88
32	Stilling et al., 2014	*Genes Brain and Behavior*	3.661	2.4	Microbial genes, brain & behaviour – epigenetic regulation of the gut-brain axis	Review	416	37.82
33	Sandler et al., 2000	*Journal of Child Neurology*	1.134	2.0	Short-term benefit from oral vancomycin treatment of regressive-onset autism	Human study	396	15.84
34	Sgritta et al., 2019	*Neuron*	14.415	14.7	Mechanisms underlying microbial-mediated changes in social behavior in mouse models of autism spectrum disorder	Animal study	389	64.83
35	Song et al., 2004	*Applied and Environmental Microbiology*	3.81	3.9	Real-time PCR quantitation of clostridia in feces of autistic children	Human study	388	18.48
36	Kim et al., 2017	*Nature*	41.577	50.5	Maternal gut bacteria promote neurodevelopmental abnormalities in mouse offspring	Animal study	385	48.13
37	Hoban et al., 2016	*Translational Psychiatry*	4.73	5.8	Regulation of prefrontal cortex myelination by the microbiota	Animal study	383	42.56
38	Tomova et al., 2015	*Physiology & Behavior*	2.461	2.4	Gastrointestinal microbiota in children with autism in Slovakia	Human study	372	37.20
39	Petra et al., 2015	*Clinical Therapeutics*	2.925	3.2	Gut-microbiota-brain axis and its effect on neuropsychiatric disorders with suspected immune dysregulation	Review	362	36.20
40	Akdis, 2021	*Nature Reviews Immunology*	108.555	67.7	Does the epithelial barrier hypothesis explain the increase in allergy, autoimmunity and other chronic conditions?	Review	356	89.00
41	Barko et al., 2018	*Journal of Veterinary Internal Medicine*	2.286	2.1	The gastrointestinal microbiome: a review	Review	347	49.57
42	Luczynski et al., 2016	*International Journal of Neuropsychopharmacology*	Nil	4.5	Growing up in a bubble: using germ-free animals to assess the influence of the gut microbiota on brain and behavior	Review	344	38.22
43	Kang et al., 2019	*Scientific Reports*	3.998	3.8	Long-term benefit of microbiota transfer therapy on autism symptoms and gut microbiota	Human study	337	56.17
44	Vuong and Hsiao, 2017	*Biological Psychiatry*	11.984	9.6	Emerging roles for the gut microbiome in autism spectrum disorder	Review	330	41.25
45	Ghaisas et al., 2016	*Pharmacology & Therapeutics*	11.127	12.0	Gut microbiome in health and disease: linking the microbiome-gut-brain axis and environmental factors in the pathogenesis of systemic and neurodegenerative diseases	Review	325	36.11
46	Vuong et al., 2017	*Annual Review of Neuroscience*	14.675	12.1	The microbiome and host behavior	Review	319	39.88
47	Dinan et al., 2015	*Journal Of Psychiatric Research*	4.465	3.7	Collective unconscious: how gut microbes shape human behavior	Review	319	31.90
48	Williams et al., 2011	*PLoS One*	4.092	2.9	Impaired carbohydrate digestion and transport and mucosal dysbiosis in the intestines of children with autism and gastrointestinal disturbances	Human study	313	22.36
49	de Theije et al., 2014	*Brain Behavior and Immunity*	5.889	8.8	Altered gut microbiota and activity in a murine model of autism spectrum disorders	Animal study	284	25.82
50	Wang et al., 2011	*Applied And Environmental Microbiology*	3.829	3.9	Low relative abundances of the mucolytic bacterium *Akkermansia muciniphila* and *Bifidobacterium* spp. In feces of children with autism	Human study	282	20.14
51	Williams et al., 2012	*mBio*	5.625	5.1	Application of novel PCR-based methods for detection, quantitation, and phylogenetic characterization of *Sutterella* species in intestinal biopsy samples from children with autism and gastrointestinal disturbances	Human study	279	21.46
52	Moloney et al., 2014	*Mammalian Genome*	3.068	2.7	The microbiome: stress, health and disease	Review	274	24.91
53	Hoyles et al., 2018	*Microbiome*	10.465	13.8	Microbiome-host systems interactions: protective effects of propionate upon the blood-brain barrier	Human study	266	38.00
54	Wang et al., 2013	*Molecular Autism*	5.486	6.2	Increased abundance of *Sutterella* spp. and *Ruminococcus torques* in feces of children with autism spectrum disorder	Human study	261	21.75
55	Li et al., 2017	*Frontiers in Cellular Neuroscience*	4.3	4.2	The gut microbiota and autism spectrum disorders	Review	255	31.88
56	Wang et al., 2012	*Digestive Diseases and Sciences*	2.26	2.5	Elevated fecal short chain fatty acid and ammonia concentrations in children with autism spectrum disorder	Human study	252	19.38
57	Mangiola et al., 2016	*World Journal of Gastroenterology*	3.365	4.3	Gut microbiota in autism and mood disorders	Review	237	26.33
58	Liu S. M. et al., 2019	*Scientific Reports*	3.998	3.8	Altered gut microbiota and short chain fatty acids in Chinese children with autism spectrum disorder	Human study	235	39.17
59	Sherwin et al., 2019	*Science*	41.846	44.8	Microbiota and the social brain	Review	227	37.83
60	Yap et al., 2010	*Journal of Proteome Research*	5.113	3.8	Urinary metabolic phenotyping differentiates children with autism from their unaffected siblings and age-matched controls	Human study	224	14.93
61	Borre et al., 2014a	*Advances in Experimental Medicine and Biology*	1.958	3.65	The impact of microbiota on brain and behavior: mechanisms & therapeutic potential	Review	221	20.09
62	Lees et al., 2013	*Journal of Proteome Research*	5.001	3.8	Hippurate: the natural history of a mammalian-microbial cometabolite	Review	220	18.33
63	Pärtty et al., 2015	*Pediatric Research*	2.761	3.1	A possible link between early probiotic intervention and the risk of neuropsychiatric disorders later in childhood: a randomized trial	Human study	212	21.20
64	Cenit et al., 2017	*World Journal of Gastroenterology*	3.3	4.3	Influence of gut microbiota on neuropsychiatric disorders	Review	207	25.88
65	Cristofori et al., 2021	*Frontiers in Immunology*	8.787	5.7	Anti-inflammatory and immunomodulatory effects of probiotics in gut inflammation: a door to the body	Review	205	51.25
66	Wang et al., 2016	*Journal of Neurogastroenterology and Motility*	2.457	3.3	Effect of probiotics on central nervous system functions in animals and humans: a systematic review	Review	205	22.78
67	Golubeva et al., 2017	*EBioMedicine*	6.183	9.7	Microbiota-related changes in bile acid & tryptophan metabolism are associated with gastrointestinal dysfunction in a mouse model of autism	Animal study	204	25.50
68	Mayer et al., 2014b	*BioEssays*	4.73	3.2	Altered brain-gut axis in autism: comorbidity or causative mechanisms?	Review	204	18.55
69	Knight et al., 2017	*Annual Review of Genomics and Human Genetics*	8.676	7.7	The microbiome and human biology	Review	203	25.38
70	Zhu et al., 2020	*Journal of Neuroinflammation*	8.322	9.3	The progress of gut microbiome research related to brain disorders	Review	199	39.80
71	Fattorusso et al., 2019	*Nutrients*	4.546	4.8	Autism spectrum disorders and the gut microbiota	Review	197	32.83
72	Diaz-Gerevini et al., 2016	*Nutrition*	3.42	3.2	Beneficial action of resveratrol: how and why?	Review	195	21.67
73	Evrensel and Ceylan, 2015	*Clinical Psychopharmacology and Neuroscience*	1.5	2.4	The gut-brain axis: the missing link in depression	Review	195	19.50
74	Altves et al., 2020	*Bioscience of Microbiota Food and Health*	3.121	2.5	Interaction of the microbiota with the human body in health and diseases	Review	193	38.60
75	Gacias et al., 2016	*eLife*	7.725	6.4	Microbiota-driven transcriptional changes in prefrontal cortex override genetic differences in social behavior	Animal study	190	21.11
76	Kang et al., 2018	*Anaerobe*	2.704	2.5	Differences in fecal microbial metabolites and microbiota of children with autism spectrum disorders	Human study	189	27.00
77	Luna et al., 2017	*Cellular and Molecular Gastroenterology and Hepatology*	Nil	7.1	Distinct microbiome-neuroimmune signatures correlate with functional abdominal pain in children with autism spectrum disorder	Human study	189	23.63
78	Sherwin et al., 2018	*Annals of the New York Academy of Sciences*	4.295	4.1	Recent developments in understanding the role of the gut microbiota in brain health and disease	Review	186	26.57
79	Vendrik et al., 2020	*Frontiers in Cellular and Infection Microbiology*	5.293	4.6	Fecal microbiota transplantation in neurological disorders	Review	183	36.60
80	Long-Smith et al., 2020	*Annual Review of Pharmacology and Toxicology*	13.82	11.2	Microbiota-gut-brain axis: new therapeutic opportunities	Review	183	36.60
81	Groer et al., 2014	*Microbiome*	Nil	13.8	Development of the preterm infant gut microbiome: a research priority	Review	183	16.64
82	Srikantha and Mohajeri, 2019	*International Journal of Molecular Sciences*	4.556	4.9	The possible role of the microbiota-gut-brain-axis in autism spectrum disorder	Review	179	29.83
83	Hiippala et al., 2016	*Frontiers in Microbiology*	4.076	4.0	Mucosal prevalence and interactions with the epithelium indicate commensalism of *Sutterella* spp.	Human study	179	19.89
84	Nishino et al., 2013	*Neurogastroenterology and Motility*	3.424	3.5	Commensal microbiota modulate murine behaviors in a strictly contamination-free environment confirmed by culture-based methods	Animal study	178	14.83
85	Grimaldi et al., 2018	*Microbiome*	10.465	13.8	A prebiotic intervention study in children with autism spectrum disorders	Human study	176	25.14
86	Dinan and Cryan, 2015	*Current Opinion in Clinical Nutrition and Metabolic Care*	4.033	3.0	The impact of gut microbiota on brain and behaviour: implications for psychiatry	Review	176	17.60
87	Mulle et al., 2013	*Current Psychiatry Reports*	3.054	5.5	The gut microbiome: a new frontier in autism research	Review	174	14.50
88	Burokas et al., 2015	*Advances in Applied Microbiology*	4.128	5.515	Microbiota regulation of the mammalian gut-brain axis	Review	173	17.30
89	Holmes et al., 2012	*Science Translational Medicine*	10.757	15.8	Therapeutic modulation of microbiota-host metabolic interactions	Review	171	13.15
90	Matta et al., 2019	*Brain Behavior and Immunity*	6.633	8.8	The influence of neuroinflammation in autism spectrum disorder	Review	170	28.33
91	Dinan and Cryan, 2017a	*Psychosomatic Medicine*	3.81	2.9	Brain-gut-microbiota axis and mental health	Review	170	21.25
92	Newell et al., 2016	*Molecular Autism*	4.833	6.2	Ketogenic diet modifies the gut microbiota in a murine model of autism spectrum disorder	Animal study	169	18.78
93	Sherwin et al., 2016	*CNS Drugs*	4.394	7.4	May the force be with you: the light and dark sides of the microbiota-gut-brain axis in neuropsychiatry	Review	168	18.67
94	Liu et al., 2015	*Journal of Agricultural and Food Chemistry*	Nil	5.7	Modulation of gut microbiota brain axis by probiotics, prebiotics, and diet	Review	167	16.70
95	Chen et al., 2021	*Nutrients*	6.706	4.8	Regulation of neurotransmitters by the gut microbiota and effects on cognition in neurological disorders	Review	165	41.25
96	Jyonouchi et al., 2002	*Neuropsychobiology*	2.065	2.3	Innate immunity associated with inflammatory responses and cytokine production against common dietary proteins in patients with autism spectrum disorder	Human study	164	7.13
97	Choi and Cho, 2016	*Clinical Endoscopy*	Nil	2.1	Fecal microbiota transplantation: current applications, effectiveness, and future perspectives	Review	163	18.11
98	Ming et al., 2012	*Journal of Proteome Research*	5.056	3.8	Metabolic perturbance in autism spectrum disorders: a metabolomics study	Human study	163	12.54
99	Liu F. T. et al., 2019	*Translational Psychiatry*	5.28	5.8	Altered composition and function of intestinal microbiota in autism spectrum disorders: a systematic review	Review	162	27.00
100	Spielman et al., 2018	*Neurochemistry International*	3.994	4.4	Unhealthy gut, unhealthy brain: the role of the intestinal microbiota in neurodegenerative diseases	Review	161	23.00

### Citations

The total citation frequency for all the 100 selected articles was 40,662, with a median citation of 280.5. The number of citations for each article ranged from 161 to 2,081. The top article with the most total citations was “Microbiota modulate behavioral and physiological abnormalities associated with neurodevelopmental disorders” by Hsiao et al. (2013) (total citations of 2,081). The most-cited human study was “Microbiota transfer therapy alters gut ecosystem and improves gastrointestinal and autism symptoms: an open-label study” by Kang et al. (2017) (total citations of 722), while the most cited randomized clinical trial was “A possible link between early probiotic intervention and the risk of neuropsychiatric disorders later in childhood: a randomized trial” by Pärtty et al. (2015) (total citations of 212).

**FIGURE 3:**
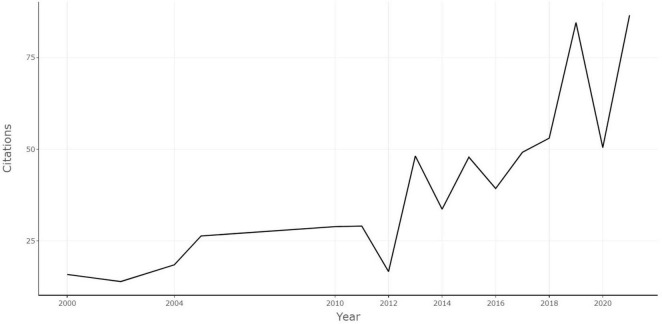
The trends of the annual citations of the 100 most-cited articles.

To exclude the effect of year on citation numbers, the annual citation rate was analyzed. Figure 3 demonstrates the trends of citations per year of the 100 selected articles. The annual citation rate was trending upward overall from 2000 to 2021. The annual citation was 15.8 in 2000, and it reached the peak in 2021 with annual citations of 86.6. The annual citation rate of each paper ranged from 7.13 to 294.83. The top article with the most annual citations was “The microbiota-gut-brain axis” by Cryan et al. (2019) (annual citations of 294.83).

**FIGURE 4:**
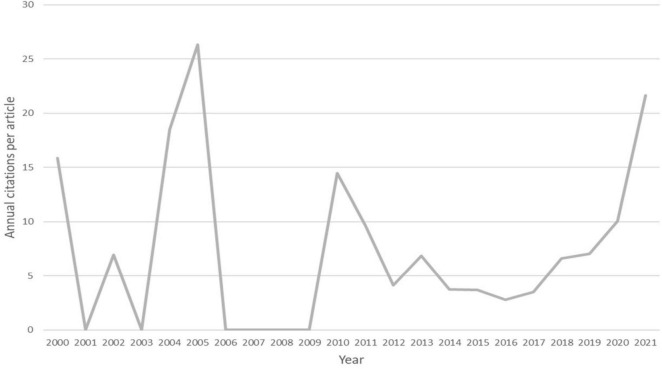
The trends of the annual citations per article of the 100 most-cited articles.

To better understand the relationship between annual citations and annual publications, the average number of citations per article per year was calculated. Figure 4 presents this average citation count for each year. The trend was fluctuating prior to 2013, as there were fewer than five articles published each year. However, as the annual number of publications increased in recent years, the trend showed an overall upward trajectory.

The total citation rate of an article was not significantly correlated to the year of publication (*r* = 0.097, *p* > 0.05, Spearman’s rho test). However, the annual citation rate of an article was significantly related to the year of publication (*r* = 0.481, *p* < 0.01, Spearman’s rho test).

Among the 100 selected articles, there were 11 animal studies, 27 human studies, and 62 reviews. The total citation rate and annual citation rate were not significantly different between the three study types (*p* > 0.05, Kruskal–Wallis test). When combining animal and human studies into a single category of experimental studies, the citation rates for clinical studies were still not significantly different from those of review articles (*p* > 0.05, Mann–Whitney *U* test).

### Countries

There were 28 countries involved in the 100 most-cited articles. The United States was involved in the highest number of publications (*n* = 42), followed by Ireland (*n* = 20), Italy (*n* = 9), England (*n* = 8), and China (*n* = 8). The details of the number of publications of each country are listed in Table 2.

**TABLE 2:** Number of publications of each country.

Country	Number of publications
United States	42
Ireland	20
Italy	9
England	8
China	8
Australia	6
Belgium	3
Canada	3
Finland	2
Japan	2
Netherlands	2
Sweden	2
Switzerland	2
Turkey	2
Argentina	1
Denmark	1
France	1
Germany	1
Greece	1
India	1
Israel	1
Malaysia	1
Philippines	1
Russia	1
Singapore	1
Slovakia	1
South Korea	1
Spain	1

**FIGURE 5:**
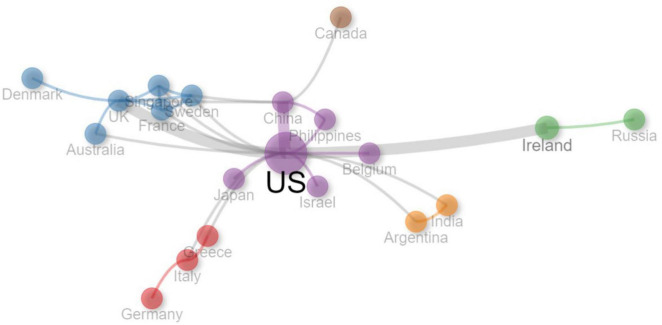
Collaboration network between countries. The color of the node represents different collaboration cluster, the width of the curved line indicates the link strength, and the distance between the nodes denotes approximate relatedness among the nodes.

There were 20 countries which had collaborations with others. Figure 5 displays the collaboration network between these countries. The color of the node in Figure 5 represents different collaboration cluster, the width of the curved line indicates the link strength, and the distance between the nodes denotes approximate relatedness among the nodes. The United States had the most collaborations with other countries, and worked closely with the United Kingdom, Ireland, and China.

### Authors

Among the 491 authors involved in the 100 most-cited articles, 8 authors published 5 or more articles. John F. Cryan was the most productive author, with 20 articles, followed by Timonthy G. Dinan with 19 articles. Table 3 lists the top 10 authors with most published articles.

**TABLE 3:** Number of publications of the top 10 authors.

Author	Number of publications
John F. Cryan	20
Timothy G. Dinan	19
Gerard Clarke	8
Sarkis K. Mazmanian	6
James B. Adams	5
Dae-Wook Kang	5
Rosa Krajmalnik-Brown	5
Eoin Sherwin	5
Elaine Y. Hsiao	4
Rob Knight	4

Most authors collaborated with others to publish their papers. However, Cezmi A. Akdis published a notable single-authored article titled “Does the epithelial barrier hypothesis explain the increase in allergy, autoimmunity and other chronic conditions?” in *Nature Reviews Immunology*. He is a professor in University of Zürich Medical Faculty and the Director of the Swiss Institute of Allergy and Asthma Research in Davos, Switzerland (Swiss Institute of Allergy and Asthma Research, 2024).

Among all the authors, Timothy G. Dinan and John F. Cryan collaborated most frequently. Timothy G. Dinan is a Professor of Psychiatry at University College Cork (University College Cork, 2024), while John F. Cryan is a Professor and Chair, Department of Anatomy and Neuroscience, University College Cork (APC Microbiome Ireland, 2024). Notably, many frequent collaborators are from the same institutions. For example, Emeran A. Mayer, the Director of the Gail and Gerald Oppenheimer Family Center for Neurobiology of Stress and a Professor of Psychology Medicine (UCLA Brain Research Institute, 2024), and Kirsten Tillisch, a Professor of Medicine and gastroenterologist with a clinical interest in chronic pain and functional gastrointestinal disorders (UCLA Health, 2024), are both based at the University of California, Los Angeles, United States. Likewise, Elaine Holmes and Jeremy K. Nicholson are affiliated with the Faculty of Medicine at Imperial College London, United Kingdom. Elaine Holmes is a Professor of Chemical Biology with research interests in discovering and developing metabolic biomarkers for disease in personalized healthcare and population studies (Imperial College London, 2024a). Jeremy K. Nicholson, an Emeritus Professor of Biological Chemistry, focuses on personalized healthcare through metabolic phenotyping and systems medicine (Imperial College London, 2024b). All these researchers contributed to their collaborative works with their unique expertise and a shared focus on interactions between the brain, gut, and microbiome. The research area of autism and gut microbiota brought together experts from diverse disciplines, including psychiatry, neuroscience, gastroenterology, biological chemistry, and chemical biology.

**FIGURE 6:**
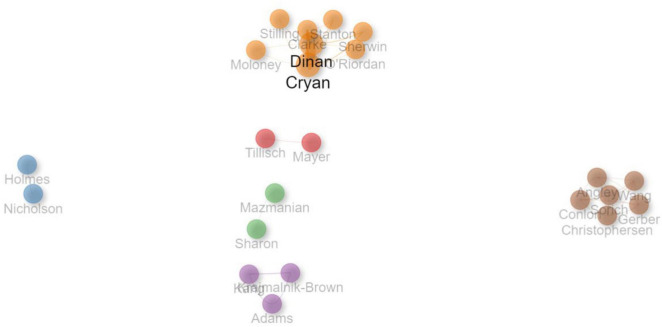
Collaboration network between authors. The color of the node represents different collaboration cluster, the width of the curved line indicates the link strength, and the distance between the nodes denotes approximate relatedness among the nodes.

Figure 6 illustrates the collaboration network between the authors who had at least three collaborations. The color of the node represents different collaboration cluster, the width of the curved line indicates the link strength, and the distance between the nodes denotes approximate relatedness among the nodes.

### Journals

The 100 selected articles were published in 76 journals. The latest impact factors of the journals ranged from 2.0 to 69.2. *Nature Reviews Microbiology* was the journal with the highest impact factor, and published the article “The gut microbiota-brain axis in behaviour and brain disorders.” *Journal of Child Neurology*, with the lowest impact factor (impact factor of 1.134 at the time of publication and impact factor of 2 in 2023), published the paper “Short-term benefit from oral vancomycin treatment of regressive-onset autism.” Among the 76 journals, 14 published at least 2 of the selected articles. *Microbiome* was the most productive journal, with five articles, followed by *Cell* with four articles. *Brain Behavior and Immunity, Frontiers in Microbiology, Journal of Proteome Research, Nutrients*, and *PLoS One* all published three articles. The number of publications of the 100 most-cited articles in that journal was not significantly related to the journal’s latest impact factor (*r* = 0.016, *p* > 0.05, Spearman’s rho test). The list of journals with at least two publications is presented in Table 4.

**TABLE 4:** Journals with at least two publications.

Name of journal	Number of articles	Journal impact factor in 2023
*Microbiome*	5	13.8
*Cell*	4	45.6
*Brain Behavior and Immunity*	3	8.8
*Frontiers in Microbiology*	3	4.0
*Journal of Proteome Research*	3	3.8
*Nutrients*	3	4.8
*PLoS One*	3	2.9
*Anaerobe*	2	2.5
*Applied and Environmental Microbiology*	2	3.9
*International Journal of Molecular Sciences*	2	4.9
*Molecular Autism*	2	6.2
*Scientific Reports*	2	3.8
*Translational Psychiatry*	2	5.8
*World Journal of Gastroenterology*	2	4.3

### KeyWords Plus

**FIGURE 7:**
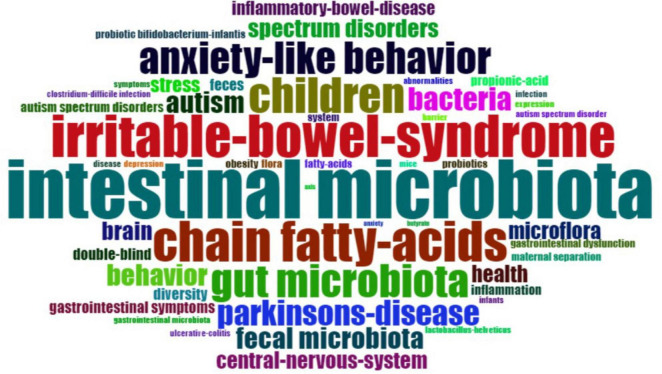
The most-used KeyWords Plus. The font size is proportional to the frequency of the word.

**FIGURE 8:**
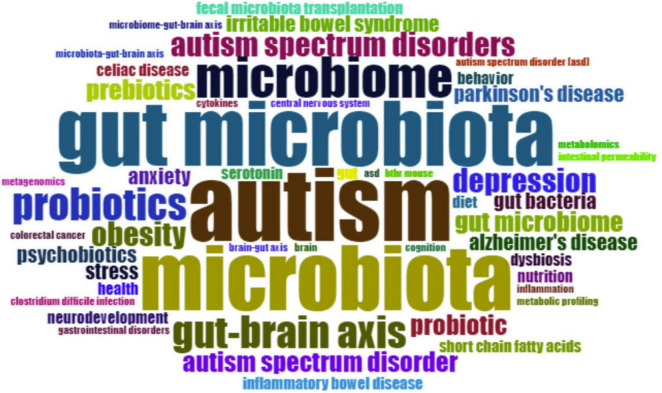
The most-used authors’ keywords. The font size is proportional to the frequency of the word.

KeyWords Plus refer to indexed keywords derived from the titles of referenced articles that occur at least twice in the bibliography, and they offer valuable insights into research trends (Tomaszewski, 2023). Figure 7 provides an overview of the most-used KeyWords Plus. The most popular KeyWords Plus were “intestinal microbiota” (*n* = 35), “irritable bowel syndrome” (*n* = 25), and “chain fatty acids” (*n* = 24). To better understand the development of KeyWords Plus, the frequency of the original authors’ keywords was analyzed. Figure 8 presents an overview of the frequency of authors’ keywords, where larger font sizes indicate higher frequencies. In comparison to the authors’ keywords, certain KeyWords Plus, such as “irritable bowel syndrome” and “chain fatty acids,” appeared more frequently, highlighting emerging trends in this field. It was also observed that some KeyWords Plus, such as “obesity” and “probiotics” were used less frequently than the authors’ keywords.

**FIGURE 9:**
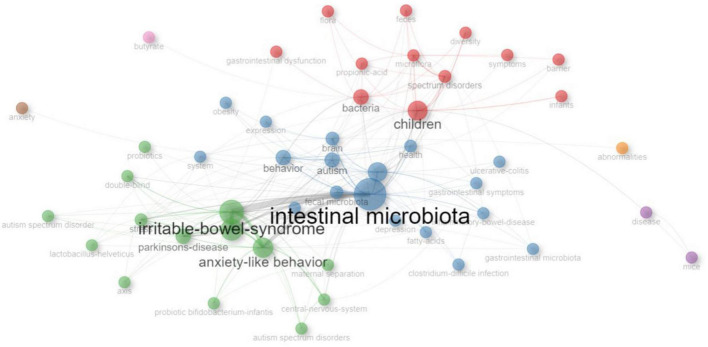
The co-occurrence network of KeyWords Plus. The size of the node is proportional to the frequency of the word, the color of the node represents different cluster, and the width of the curved line indicates the link strength.

Figure 9 illustrates the co-occurrence network of KeyWords Plus. The size of the node is proportional to the frequency of the word, the color of the node represents different cluster, and the width of the curved line indicates the link strength. The most popular word “intestinal microbiota” was linked to many other words, such as “autism,” “brain,” “children,” and “irritable bowel syndrome.”

### Thematic analysis

**FIGURE 10:**
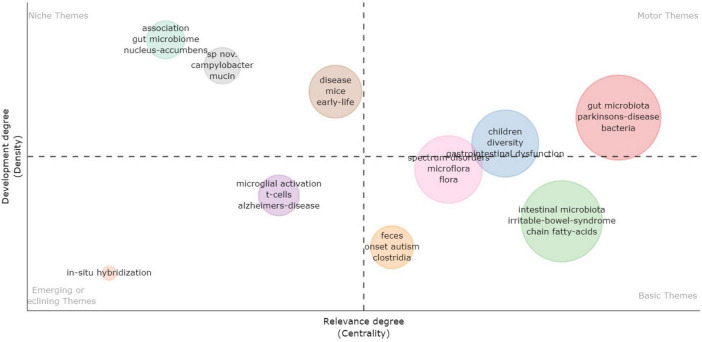
Thematic map analysis based on KeyWords Plus. Upper-right quadrant: motor themes – high density (developed) and high centrality (relevant); upper-left quadrant: niche themes – high density (developed) and low centrality (less relevant); lower-left quadrant: emerging or declining themes – low density (less developed) and low centrality (less relevant); lower-right quadrant: basic themes – low density (less developed) and high centrality (relevant).

The thematic map analysis based on KeyWords Plus is illustrated in Figure 10. A thematic map allows four typologies of themes to be categorized based on their placement in specific quadrants. Themes in the upper-right quadrant are identified as motor themes, characterized by both high density and centrality, signifying their development and relevance in the research field. In the upper-left quadrant, themes are classified as niche themes, marked by high density but low centrality, indicating their isolated development. Themes in the lower-left quadrant have low centrality and density, suggesting they are weakly developed and marginal. In the lower-right quadrant are basic themes, featuring high centrality (relevance) and low density (less development). As illustrated in Figure 8, several pertinent themes are discernible in this research field, such as chain-fatty acids, bacteria, and irritable bowel syndrome (IBS).

## Discussion

### General information

This study combined bibliometric analysis with network visualization to identify the first 100 highly impactful manuscripts in the field of ASD and gut microbiota, based on global citation frequency. It highlights the contributions that have driven substantial progress in this field, identifies the current research trends, and provides guidance for future research directions. Various aspects in this research domain were explored, including the top articles with most citations, correlations between citation rates and publication time, distribution of involved countries, contributions of key authors, impactful journals with most publications, and relevant themes in this field.

Among the 100 most-cited articles in the current review, the average number of citations annually increased over the years and was significantly correlated to the year of publication. However, there was no significant association between total number of citations and time of publication. These trends are consistent with bibliometric analyses in other areas, such as burns (Ring et al., 2020) and insomnia (Wan et al., 2022). This is likely due to the tendency of total citations to favor older publications, as more recent papers have a shorter duration to accumulation citations. The average number of citations annually, as different from the total citations, can eliminate the effect of time on citation numbers and provide a more accurate view of the immediate impact of the articles. Besides, as the association between autism and gut microbiota is a rapidly evolving field (Wang Q. et al., 2023), newer studies in this research area often receive high initial attention and are cited more frequently within the initial years, as they may represent cutting-edge findings or novel methodologies, leading to an increase in annual citation averages over time. The area in autism and gut microbiota may be different from foundational research which tends to accumulate citations consistently over long periods and has obscured direct association with the time of publication. Overall, the quantity of citations of an article is a useful proxy to indicate the significant of the study (Landreneau et al., 2020). It can be implied that the influence of research in autism and gut microbiota has been steadily increasing over the years.

In terms of the distribution of countries, the United States contributed to the largest volume of the publications, followed by other countries, such as Ireland, England, and China. These findings are similar to bibliometric reviews in other conditions, such as schizophrenia (Yang et al., 2022) and intellectual disability (Ying et al., 2022). The United States holds a competitive edge in this research domain and is likely to have a significant impact on the direction of research in this field and maintain the most robust global collaborations. The information of the distribution of countries can be valuable for researchers seeking to choosing the most suitable place for additional training or collaborative opportunities.

Each journal contributed one to five of the 100 most-cited articles. The number of the articles that the journal contributed was not related to the impact factor of the journal. These findings are consistent with other review papers on impactful studies, such as the landmark studies in burns (Ring et al., 2020). The impact factor was first introduced by Garfield (2006) and was commonly used as a measure to indicate the significance of a journal within its respective field. The impact factor pertains exclusively to journals and does not extend to individual articles. Thus, it is possible for a highly impactful study to be published in a journal with low impact factor. In the current review, one of the highly cited papers “short-term benefit from oral vancomycin treatment of regressive-onset autism” was published in 2000 in *Journal of Child Neurology*, a journal with an impact factor of 1.134 at the time of publication. At that time this article was submitted to a low impact journal was likely due to several reasons. This article focused on short-term effects in a small sample with preliminary nature of findings (Sandler et al., 2000) and this could make high-impact journal hesitant to publish it. Besides, at that time the concept of a link between autism and gut microbiota was not widely accepted, especially in high-impact journals. In addition, the authors at that time might not be aware of the significance of their work or chose the journal based on the audience specialization and journal readership. Nonetheless, this article is one of the early works to suggest a potential link between gut microbiota and autism and is one of the foundational references for researchers exploring this area. It has subsequently cited by numerous papers published in high-impact journals, such as *Nature Reviews Gastroenterology & Hepatology* (Hung and Margolis, 2024), *Clinical Microbiology Reviews* (Yadegar et al., 2024), and *Microbiome* (LaPelusa et al., 2021).

Our findings share certain similarities with other bibliometric analyses on gut microbiota in various conditions. For example, a bibliometric analysis on gut microbiota and Parkinson’s disease identified similar main research topics, including “short-chain fatty acids,” “probiotics,” and ‘inflammation” (Li et al., 2024). Another similar analysis on gut microbiota and obesity found similar top journals in this area, such as *Nutrients*, *Scientific Reports*, and *Frontiers in Microbiology* (Wang M. et al., 2024). Various bibliometric analyses related to gut microbiota revealed an overall upward trend in the number of publications and the United States being one of the leading countries in those research fields (Li et al., 2024; Ouyang et al., 2024; Wang M. et al., 2024). Together with other studies on similar fields of investigation, this current study can offer a clear insight into the current research landscape and emerging trends, serving as a valuable reference for researchers entering this field of gut microbiota.

### Influential studies

The most-cited article among all the selected papers was “Microbiota modulate behavioral and physiological abnormalities associated with neurodevelopmental disorders” by Hsiao et al. (2013) published in *Cell*. This is a landmark study, as it demonstrated gastrointestinal barrier defects and microbiota changes in the maternal immune activation mouse model with autistic symptoms. This study found that treatment with the human commensal *Bacteroides fragilis* corrected gut permeability defects, altered the composition of the microbiota, regulated the serum levels of the metabolite of 4-ethylphenylsulfate, and alleviated abnormal communicative, anxiety-like, stereotyped, and sensorimotor behaviors. It proposed a groundbreaking idea that ASD could be potentially a disorder related to the gut, and that therapies involving the microbiome might offer a safe and effective approach to treating the disorder.

The human study with most citations was “Microbiota transfer therapy alters gut ecosystem and improves gastrointestinal and autism symptoms: an open-label study” authored by Kang et al. (2017) and published in *Microbiome*. In this open-label clinical trial, the efficacy of Microbiota Transfer Therapy was evaluated in terms of its impact on gut microbiota, gastrointestinal and autistic symptoms in children diagnosed with ASD. This study found that the abundance of *Bifidobacterium, Prevotella*, and *Desulfovibrio* increased after the intervention and the improvement persisted till the end of 8 weeks follow-up. These findings are promising and represent a pivotal advancement in understanding the relationship between gut microbiota and ASD.

The randomized clinical trial among the 100 most-cited articles was performed by Pärtty et al. (2015) who wrote the article “A possible link between early probiotic intervention and the risk of neuropsychiatric disorders later in childhood: a randomized trial” published in *Pediatric Research*. In this clinical trial, 75 infants were randomized to receive *Lactobacillus rhamnosus* GG or placebo during their first 6 months of life and were followed up for 13 years. At 13 years old, Asperger syndrome or attention deficit hyperactivity disorder was diagnosed in 17.1% of children in the placebo group, while none in the probiotic group. This influential study demonstrated, for the first time, that certain probiotics could potentially mitigate the risk of developing specific neurodevelopmental disorders.

The impactful paper published in the journal with the lowest impact factor among all the included articles was “Short-term benefit from oral vancomycin treatment of regressive-onset autism” by Sandler et al. (2000) published in *Journal of Child Neurology*. This open-label clinical trial demonstrated the short-term improvement in autistic symptoms after oral vancomycin treatment among 11 children with regressive-onset ASD. This early study, published in July 2000, indicated the potential existence of a gut-brain connection in a subgroup of children with both ASD and diarrhea.

### Future outlook

The co-occurrence network of KeyWords Plus and thematic analysis in this study identified several important hotspots and future directions in this research area, such as microbial metabolites of short-chain fatty acids (SCFAs), role of bacteria, and overlaps of IBS.

Short-chain fatty acids are monocarboxylic acids containing fewer than six carbon atoms (Schonfeld and Wojtczak, 2016). The majority of SCFAs in the human intestine are acetic acid, butyric acid and propionic acid (Iniguez-Gutierrez et al., 2020). These organic acids result from the fermentation of dietary fiber and resistant starch in the intestine (Portincasa et al., 2022). Several well-designed animal studies have been performed to explore the relationship between SCFAs and ASD. For example, one study in Canada found that rats treated with propionic acid displayed more stereotypic behavior, nose pokes and locomotive activity (Meeking et al., 2020). Studies in human participants have also reported changes in SCFAs in the stool of ASD subjects. One recent study revealed that children with ASD and constipation had excessive propionic acid in feces (He et al., 2023). This study provided new clues to understand the etiology and biomarkers for ASD. However, the results in human studies are inconsistent (Lagod and Naser, 2023). The variability in human study outcomes highlights the need for further research on SCFA levels in individuals with ASD.

The composition of bacteria in the human gastrointestinal tract is complex. There are still inconsistences regarding the association between different bacteria and ASD in different studies. Some studies have reported higher abundance of *Lactobacillus* in ASD (Pulikkan et al., 2018; Strati et al., 2017), while it is also reported that *Lactobacillus* has decreased levels (Iovene et al., 2017). The diversity of gut microbiota has been reported to be either increased (Coretti et al., 2017) or decreased (Dan et al., 2020) in persons with ASD. Besides, the ratio between *Firmicutes* and *Bacteroidetes* in persons with ASD has been reported higher in some studies (Strati et al., 2017), and lower in other studies (Zhang et al., 2018). It has been pointed out that the inconsistent conclusions between different studies are likely due to various reasons, including underpowered research design and variation in use of multiple testing corrections (Li et al., 2022). In addition, gut microbiota composition may also be affected by other factors, such as age, body mass index, and dietary habits (Rinninella et al., 2019). Future investigations with more comprehensive and standardized methods may shed light on the intricate connections linking gut bacteria and ASD.

Irritable bowel syndrome is a chronic gastrointestinal disease with the core clinical symptoms of recurrent abdominal discomfort or pain, and altered bowel habits (Huang et al., 2023). IBS is commonly observed as a comorbid condition in individuals with ASD (Penzol et al., 2019). In conditions such as IBS and ASD, where dysbiosis is potentially present, the utilization of prebiotics and probiotics may serve as a low-risk therapeutic approach to improve symptoms (Abdellatif et al., 2020). One recent pilot randomized clinical trial published in *Cell Host & Microbe* tested the effect of bacterial species *Limosilactobacillus reuteri* in children with ASD and found that the bacteria significantly improved the social functioning (Mazzone et al., 2024). Results in recent studies are compelling to encourage additional future research on utilizing probiotics or specific microbes as treatment options for persons with ASD.

### Limitations

Although this study provides valuable and comprehensive insights to help researchers and policymakers to understand the research trends and guide feature decision-making, it has several limitations. First, the literature search was conducted only in the Web of Science database. While the Web of Science is the leading database in scientometrics and many studies rely solely on it for bibliometric analysis, our findings could be more comprehensive if additional databases were included. Second, although bibliometric analysis provides a broad overview of research trends and networks, it may lack in-depth analysis as it does not evaluate the quality of the numerous studies. Third, this research field is advancing rapidly. It is possible that some recently published high-quality studies may be overlooked, due to the low accumulated citation numbers.

## Conclusion

The present study, to our knowledge, is the first bibliometric analysis to comprehensively explore the 100 most-cited articles in the field of ASD and gut microbiota. By identifying and analyzing these pivotal studies, we provide a detailed overview of the most influential research in this domain. The results highlight key trends, emerging topics, and potential future directions for investigation. This analysis not only illuminates the current landscape of research but also offers valuable insights for researchers, clinicians, and policymakers. It serves as a critical reference for guiding the development and focus of future scientific inquiries and clinical practices related to ASD and gut microbiota.

## Data Availability

The original contributions presented in this study are included in this article/supplementary material, further inquiries can be directed to the corresponding authors.
